# Effect of nitrogen atom positioning on the trade-off between emissive and photocatalytic properties of carbon dots

**DOI:** 10.1038/s41467-017-01463-x

**Published:** 2017-11-09

**Authors:** Santanu Bhattacharyya, Florian Ehrat, Patrick Urban, Roland Teves, Regina Wyrwich, Markus Döblinger, Jochen Feldmann, Alexander S. Urban, Jacek K. Stolarczyk

**Affiliations:** 1 Chair for Photonics and Optoelectronics, Department of Physics and Center for NanoScience (CeNS), Ludwig-Maximilians-Universität München, Amalienstr. 54, 80799 Munich, Germany; 2 Nanosystems Initiative Munich (NIM), Schellingstr. 4, 80799 Munich, Germany; 3 Department of Chemistry, Ludwig-Maximilians-Universität München, Butenandtstr. 5–13 (E), 81377 Munich, Germany

## Abstract

Carbon dots (CDs) are a versatile nanomaterial with attractive photoluminescent and photocatalytic properties. Here we show that these two functionalities can be easily tuned through a simple synthetic means, using a microwave irradiation, with citric acid and varying concentrations of nitrogen-containing branched polyethyleneimine (BPEI) as precursors. The amount of BPEI determines the degree of nitrogen incorporation and the different inclusion modes within the CDs. At intermediate levels of BPEI, domains grow containing mainly graphitic nitrogen, producing a high photoluminescence yield. For very high (and very low) BPEI content, the nitrogen atoms are located primarily at the edge sites of the aromatic domains. Accordingly, they attract photogenerated electrons, enabling efficient charge separation and enhanced photocatalytic hydrogen generation from water. The ensuing ability to switch between emissive and photocatalytic behavior of CDs is expected to bring substantial improvements on their efficiency for on-demand light emission or energy conversion applications.

## Abstract (web summary)

Carbon dots are an emergent class of nanomaterials that hold promise for innovations in imaging, sensing, and catalytic technologies. Here, Stolarczyk and colleagues control the nitrogen-atom content and location within carbon dots, reporting the resulting impact on emissivity and photocatalytic behaviour.

## Introduction

The appeal of carbon dots (CDs) as an emergent class of nanomaterials since their discovery in 2004^1, 2^ stems from their attractive luminescent properties, ease of fabrication and functionalization, photostability, and biocompatibility. They hold promise for widespread applications in light emission, catalysis, sensing, as well as theranostic and imaging devices as an alternative to inorganic quantum dots^3–15^. CDs possess a range of unique and rather intriguing optoelectronic properties, for example they exhibit a very large (over 100 nm) Stokes shift. In addition, in contrast to inorganic semiconductors, in most CDs the emission wavelength depends on the excitation wavelength, the reason for which is still highly debated^16–24^. CDs have a complex internal structure, which is generally said to comprise *sp* ^*2*^-hybridized aromatic domains embedded in an amorphous *sp* ^*3*^-hybridized matrix^25–27^. In this context, we have recently proposed a model of internal structure of CDs as a combination of several different polycyclic aromatic hydrocarbon molecules in which the excitation-dependent photoluminescence (PL) relies on their different absorption and emission ranges and energy transfer pathways between the molecules^27^. Therein, we showed that exciton self-trapping on stacked aromatic molecules could explain the large observed Stokes shifts for CDs in general. Recently some groups have also claimed the formation of specific fluorescent molecules inside CDs could be responsible for the high PL quantum yield (QY) seen in some CDs^28–30^. What emerges from these studies is a picture of complex interplay between the constituent parts with multiple charge and energy transfer pathways determining the photoluminescent properties^31–33^. On the basis of the precursor molecules, one would expect the presence of nitrogen atoms inside CDs^33^. Consequently, considering the effect of inclusion of nitrogen atoms in the aromatic domains would extend our understanding of the optical properties of CDs^34–36^. Nitrogen atoms can be incorporated in the CDs in several different modes^37^. First, they can substitute carbon atoms inside the poly-aromatic (graphitic) structure forming σ-bonds to the neighboring three C atoms. One electron contributes to the aromatic π-bond, whereas the fifth one enters the antibonding π^∗^ molecular orbital. Substitution of an edge site in the six- or five-member aromatic ring leads to pyridinic or pyrrolic moieties, respectively. In these configurations, the nitrogen atom contributes, respectively, one or two valence electrons to the aromatic π orbital. Finally, the nitrogen atom can be part of a pendant amine or amide group. In all cases, the nitrogen incorporation leads to substantial changes in the electronic properties of the CDs^38–43^. Similar effects were also seen in graphene oxide-based dots^44, 45^. In particular, graphitic N atoms have a strong n-doping effect due to the additional π^∗^ electrons, while pyridinic and pyrrolic N atoms have a weak p-doping character^46^. The latter effect originates from a higher electronegativity of the N atom, which pulls the electron cloud away from the C atoms^47^. Clearly, a deeper understanding of the effect and mode of N incorporation during the synthesis would be highly beneficial to tune the optoelectronic properties of the CDs to the specific task in question.

Here, we study how nitrogen is incorporated in the CDs and how the inclusion mode of the nitrogen affects the photocatalytic and photo-emissive properties of the resulting CDs by monitoring PL QY and photocatalytic hydrogen generation efficiency. Specifically, we have synthesized CDs from citric acid and branched polyethyleneimine (BPEI) by a simple microwave-assisted pyrolysis technique, as previously demonstrated^37^, and changed the content of BPEI systematically, in order to vary the nitrogen content in the reaction mixture from a dopant-like amount of 2.5 up to 35%. Interestingly, we have found that the PL QY reached a maximum for an intermediate level of BPEI. Moreover, the trend for the photocatalytic H_2_ generation perfectly anti-correlated with the PL QY, increasing for both lower and very large amounts of BPEI. We show here that the perceived trade-off between these two modes of operation can be explained in terms of varying contribution of graphitic, pyridinic, and pyrrolic nitrogen atoms in the CDs. This shows how critical the role of nitrogen is for the fate of photogenerated charges and thereby the optoelectronic properties of CDs. In addition, this presents a simple synthetic method with which to control their tunable functionality by regulating specific nitrogen atom positioning in aromatic domains inside CDs. In summary, the full understanding of the contribution of the individual components of the CDs can lead to further improvements in either luminescent or photocatalytic properties of the CDs, enhancing their widespread applicability.

## Results

### Synthesis and morphological characterization

**Fig. 1:**
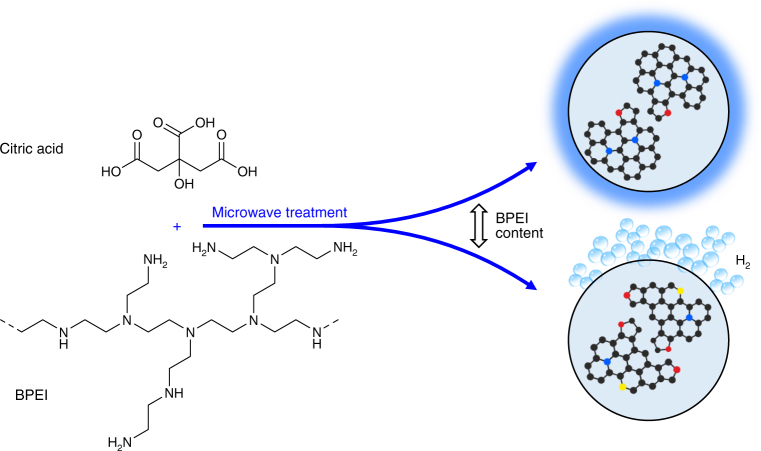
Synthesis of CDs. Schematic representation of the microwave-assisted synthesis procedure of N-doped CDs with tunable properties upon varying the concentration of the BPEI precursor (Polycyclic aromatic molecules inside the CDs with different nitrogen atom positioning, i.e., graphitic nitrogen (blue), pyrrolic nitrogen (red), and pyridinic nitrogen (yellow), respectively)

**Fig. 2:**
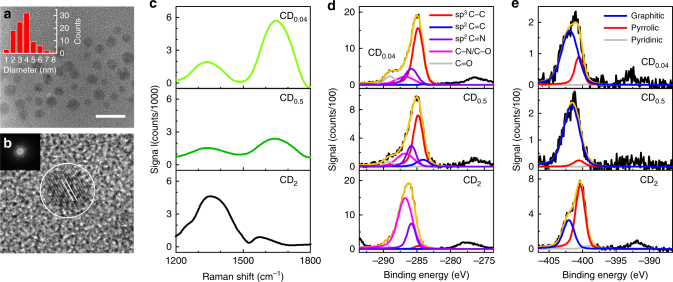
Morphological properties of CDs. **a** TEM image (inset shows the size distribution) and **b** HR-TEM image of CDs (sample: CD_0.5_) (inset shows the Fast Fourier Transformation (FFT) image). **c** Raman spectra of CDs with varying BPEI content showing the effect on D- and G-bands. De-convoluted high resolution XPS spectra for **d** carbon (C 1 s) and **e** nitrogen (N 1s) for CDs of varying BPEI content. (**a** scale bar 10 nm; **b** distance between two lattice planes marked in white lines inside the white circle represents 0.22 nm)

The CDs were prepared from citric acid as a carbon source and varying amounts of branched polyetheleneimine as a nitrogen precursor (Fig. 1). The details of the procedure are given in the Methods section^37, 48, 49^. The amount of citric acid was kept constant (1 g), while the amount of BPEI (represented throughout the manuscript as the subscript the in sample designator, CD_*x*_) was varied by a factor of 50, from 0.04 g to 2 g. Transmission electron microscopy (TEM) (Fig. 2a) confirms the formation of nearly monodisperse CDs with a diameter 3–5 nm. High-resolution (HR-) TEM images reveal prominent crystal fringes at 0.22 nm lattice spacing, consistent with the (100) plane of the graphitic crystal lattice, implying an at least partially ordered internal structure of the CDs (cf. Fig. 2b)^2, 48, 50^. The average size distribution of the CDs was nearly constant for varying amounts of BPEI (Supplementary Fig. 1).

Raman spectroscopy is a useful tool for distinguishing the ordered and disordered phases of the CDs, by investigating the occurrence of the so-called D band (~ 1350 cm^−1^) and G band (~ 1600 cm^−1^). The D band arises from the out of plane vibrations of *sp* ^2^ C in the presence of disordered states corresponding to topological *sp* ^3^ molecular defect states, while the G band results from the in plane stretching vibrations of *sp* ^2^ carbon atoms inside the aromatic domain^51^. The ratio of the peak intensities of the two bands, *I*_D_/*I*_G_, indicates the ratio of disordered and aromatic domains inside the CDs^52^. While the D band is present for all three samples (CD_0.04_, CD_0.5_, and CD_2_), the *I*_D_/*I*_G_ ratio differs markedly (cf. Fig. 2c). At lower concentration of BPEI (up to 0.5 g BPEI), I_D_/I_G_>1, indicating the prevalence of larger *sp* ^2^ aromatic domains with few defects. However, at higher concentrations of BPEI (2 g), the G band nearly vanishes, likely corresponding to more numerous *sp* ^3^ defects and thereby to a more disordered phase of CD_2_. Furthermore, a shift of the G band for CD_0.04_ and CD_0.5_ in comparison to CD_2_ can be observed. This indicates different atomic configurations (mainly of nitrogen), and molecular strain inside the aromatic domains upon changing of the BPEI concentration^53^.

X-ray photoelectron spectroscopy (XPS) measurements were performed to elucidate the bonding states of carbon and nitrogen in different CDs upon varying the BPEI concentration. As XPS probes only the surface layers of the sample, argon ion sputtering was additionally applied for 15 min to provide insight into the core structures of the CDs and the strong C 1s band at ~284–290 eV and the weaker N 1s band at ~400–404 eV were analyzed. The relative ratio of the intensity of the C 1 s and N 1 s bands correspond to the nitrogen content in the CDs and yielded nitrogen contents of ~7, ~10, and ~20% for CD_0.04,_ CD_0.5_, and CD_2_, respectively, in line with the increase in the amount of the nitrogen-containing precursor. The high resolution C 1 s spectra were de-convoluted into five different signals (cf. Fig. 2d), corresponding to carbon atoms in *sp* ^2^ C–C (284 eV), *sp* ^3^ C–C (284.8 eV), *sp* ^2^ C–N (285.8 eV), C–N/C–O (286.7 eV) and C=O (~ 289 eV) contributions^27^. For CD_0.04,_ a strong peak due to *sp* ^3^-hybridized C atoms forming a C–C bond has been observed, but only a very faint feature corresponding to *sp* ^2^-hybridized C–C bonded atoms can be discerned. At the same time, both *sp* ^2^ and *sp* ^3^ hybridized C atoms in a C–N bond have been observed very clearly, as well as the C=O bond, likely originating from both –COOH and –CONH_2_ groups. Intensities of these latter three peaks are significantly higher in the spectra for the non-sputtered samples (Supplementary Fig. 2). This is in good agreement with the expectation that the hydrophilic groups at the surface of the CDs are partially removed by the ion sputtering. Interestingly, upon increasing the BPEI concentration the *sp* ^2^ C–C bonds become stronger for CD_0.5_, presumably corresponding to the formation of larger or more numerous polycyclic aromatic domains, and then practically vanish for the CD_2_ sample. In this latter sample the sp^3^ C–C peak also strongly decreases in intensity, while the characteristic peaks of *sp* ^2^ C–N and *sp* ^3^ C–N become more prominent. This points to a larger extent of carbon-nitrogen bonding and to an increased proportion of non-conjugated *sp* ^3^ domains, corroborating the conclusions drawn from the Raman spectra.

The high resolution N 1s band can be de-convoluted into three main peaks corresponding to pyridinic (398.5 eV), pyrrolic (400.1 eV), and graphitic 400.7 eV nitrogen atoms^48^. The analysis of the spectra (cf. Fig. 2e) suggests that the graphitic N dominates in CD_0.04_ (78% of the total N signal) and in CD_0.5_ (92%), with the remaining contribution from pyrrolic nitrogen (22% and 8%, respectively). For CD_2_ the spectrum is dominated by pyrrolic nitrogen (64%), while the graphitic nitrogen contribution drops dramatically to only 33%. This value could be even lower, as a shift of the peak position from 400.7 to 401.5 eV suggests an additional contribution from amine/amide nitrogen. In addition, a small contribution from the pyridinic N can be distinguished. It appears, therefore, that the condensation reaction between the acid and the imine in the initial phase of the formation of the CDs is followed by a carbonization to produce *sp* ^2^ domains, with pyrrolic N residing at the edges of the five-member rings^41^. Further fusion of the rings leads to a substantial contribution of the graphitic nitrogen atoms. When more BPEI is available, the N-doped graphitic domains apparently dominate the CD internal structure. However, for very high BPEI contents (e.g., in CD_2_) non-aromatic *sp* ^3^-hybridized spacers, which do not convert into additional aromatic domains, become predominant. In this case, the nitrogen stays in either the pyrrolic configuration or in its original amine/amide form.

### Optical properties of carbon dots

**Fig. 3:**
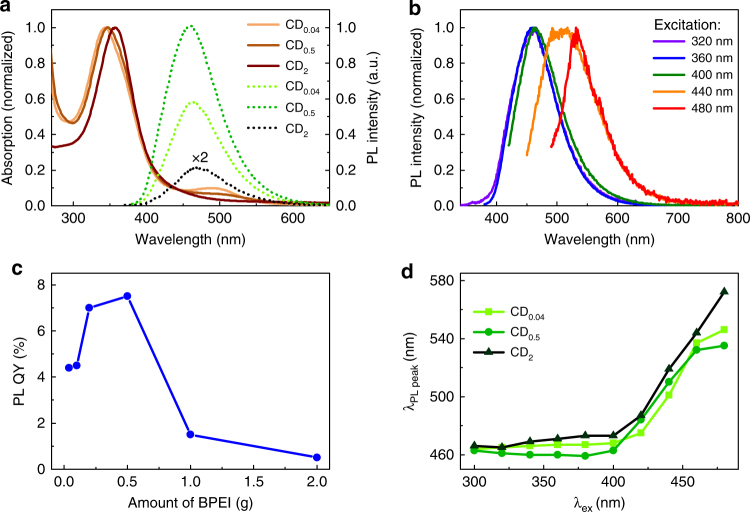
Optical properties of CDs. **a** Normalized absorption spectra (solid lines) and photoluminescence spectra (dashed lines) of three CD samples with varying BPEI content excited at λ_ex_ = 360 nm. **b** Dependence of photoluminescence quantum yield on the BPEI concentration of the CDs; **c** Normalized photoluminescence spectra excited at different wavelengths (sample: CD_0.5_). **d** Dependence of the wavelength of the photoluminescence maximum as a function of excitation wavelength for CDs with varying BPEI content

In the next step, the optical properties of the CDs were investigated as a function of the nitrogen content. The absorption spectra of all prepared CDs exhibit a prominent band at ~ 350 nm, a weak shoulder at longer wavelengths (~ 500 nm) and a strong absorption below 270 nm (Fig. 3a). The prominent band is found in nearly all CDs and is generally assigned to the π-π^∗^ transition of the aromatic hydrocarbon domains^27, 54–56^. The peak position shifts slightly from 344 to 357 nm with increasing BPEI content. This likely originates from different dielectric screening in the CDs due to varying contents of the amorphous *sp* ^3^ phase and the presence of amide and amine groups on the surface. This conjecture was corroborated by zeta potential measurements which revealed surface charges of −5, 15, and 38 mV for CD_0.04_, CD_0.5_, and CD_2_, respectively. The trend is consistent with a replacing of negatively charged carboxylic groups with positively charged ammonium groups on the CD surface. In addition, Fourier transform infrared spectroscopy (FTIR) revealed a strong C=O stretching band (1720 cm^−1^) for CD_0.04_ and CD_0.5_, which nearly vanishes in the CD_2_ sample, further supporting the notion of a change of surface functionality for CDs with varying BPEI precursor contents (Supplementary Fig. 3 and Supplementary Note 1). Moreover, the absorption shoulder at longer wavelengths decreases for increasing BPEI content. It has been previously assigned to larger polycyclic aromatic molecules, which do not seem to form for the CD_2_ sample^57–59^. The short wavelength feature likely originates from absorption by the σ-bonds and by single aromatic rings^54, 58, 59^.

To compare the PL of the CD samples, their concentration was set to yield an optical density of 0.1 (per cm) at their absorption peak around 350 nm and the samples excited at this wavelength (Fig. 3a). The PL spectra are very similar in form between the samples, with a single broad (~80 nm full-width at half maximum) emission peak centered at around 460 nm. The emission is slightly red shifted to 470 nm for the CD_2_ sample. While the form of the spectra are very similar, the intensity varies strongly, with the CD_0.5_ sample showing by far the highest PL and CD_2_ by far the lowest. To quantify this further, the PL QY of the samples was obtained using quinine sulfate in 0.1 M H_2_SO_4_ solution as a reference dye (Fig. 3b). The results show a gradual increase in the yield with increasing BPEI concentration with a maximum of 8% for CD_0.5_. Thereupon the QY promptly decreases to a value of  < 0.5% for CD_2_. To gain insight into the nature of the emitters, PL spectra were taken at different excitation wavelengths (Fig. 3c). As shown for the case of CD_0.5_, the PL spectra do not shift for short excitation wavelengths (λ_ex_), but shift strongly when excited above 400 nm. The PL of the other samples follows a similar trend (Supplementary Fig. 4). This phenomenon is commonly, although not always, observed in CDs^27^. For the ease of comparison of this shift, the emission peak position was plotted vs. the excitation wavelength for all CD samples (Fig. 3d). Herein, it becomes clearer that the PL is separated into two regions; an excitation wavelength-independent part for λ_ex_ ≤ 400 nm, and an excitation wavelength-dependent part for λ_ex_ > 400 nm. The relative PL QY also depends on excitation wavelength for all CDs (Supplementary Fig. 5). This behavior is present in all samples with only a small shift in the emission wavelength (λ_em_) between them, which suggests that there is no noticeable electronic interaction of the hydrophilic surface functional groups with the aromatic domain of CDs for our present systems. Furthermore, the measurements suggest that all samples in fact comprise almost identical emitters, but—taking into account the strongly varying PL-intensity, their numbers likely differ between the samples.

**Fig. 4:**
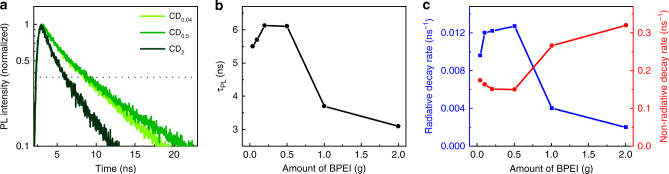
Time-resolved photoluminescence of CDs. **a** Time-resolved photoluminescence decay for different CDs (λ_ex_ = 360 nm; λ_em_ = 460 nm). **b** Photoluminescence lifetime as a function of BPEI concentration. **c** Radiative and non-radiative decay rates for CDs synthesized with different BPEI concentrations

The lifetime of photoexcited charges can provide additional information on the transfer pathways in the material. To this end, time-resolved fluorescence measurements were taken via time-correlated single photon counting with λ_ex_=360 nm and λ_em_=460 nm (Fig. 4a). For all samples the PL does not decay exponentially, but rather requires multiple exponential functions to reproduce. As the decay pathways are unclear, with many different de-excitation mechanisms possible (PL emission, non-radiative decay, energy transfer, charge transfer,…) instead of using a multiexponential fit, we have evaluated the PL decay by comparing the time at which the PL intensity has fallen to 1/e of the initial value (dashed line) and used this value as the fluorescence lifetime, τ_PL_. While the PL decay of the CD_0.04_ and CD_0.5_ samples is nearly identical, the CD_2_ sample shows a much faster decay. The PL lifetime increases initially with increasing BPEI content to a maximum of 6.1 ns around BPEI values of 0.2–0.5 and then decreases with further increasing BPEI content (Fig. 4b) down to 3.2 ns. Interestingly, the PL lifetime follows a similar trend to the PL QY shown in Fig. 3b. The close resemblance between the PL QY and PL lifetime profiles indicates that changes to both the radiative and the non-radiative recombination rates are responsible for the differences between the samples. Both rates can be calculated from the QY and lifetime results, by solving system of two equations: τ_PL_^−1^ = τ_r_^−1^ + τ_nr_^−1^ and QY = τ_nr_ / (τ_r_ + τ_nr_), where τ_r_^−1^ and τ_nr_^−1^ denote the radiative and non-radiative recombination rates, respectively^75^. The radiative decay rate exhibits a similar behavior to both the PL lifetime and PL QY, while the non-radiative decay rate shows an inverse behavior (Fig. 4c). As can be surmised from the lifetime profile, the non-radiative rate is smallest for CD_0.5_, but increases both for higher and for lower concentrations of the nitrogen precursor. On the other hand, the radiative rate reaches its maximum for CD_0.5_, in line with the QY results.

### Photocatalytic hydrogen generation with carbon dots

**Fig. 5:**
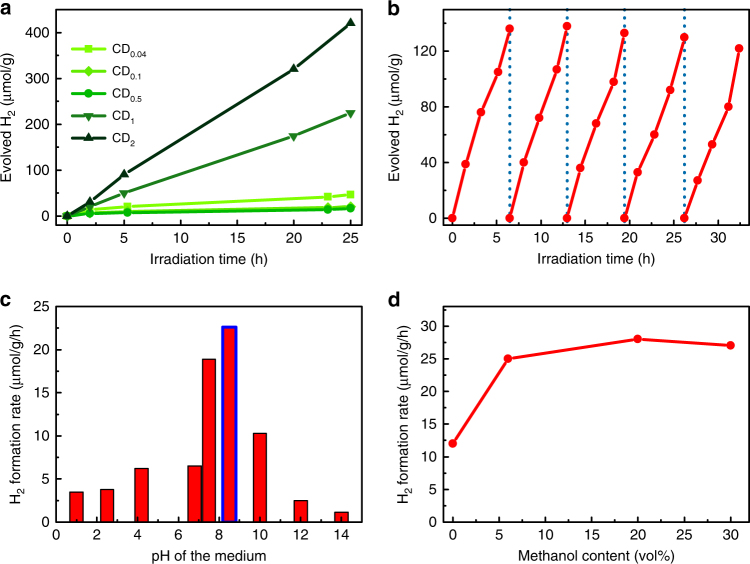
Photocatalytic hydrogen generation. **a** Hydrogen production for different CDs under Xe lamp irradiation; **b** Five cycles of hydrogen production by CD_2_. The reaction vessel was evacuated at the times denoted by the dashed blue lines; **c** Effect of pH on the hydrogen generation rate using the sample CD_2_ (column with a blue border represents the pH of the stock solution); **d** Effect of hole scavenger (methanol) concentration on the photocatalytic hydrogen generation rate

A high PL relies on keeping charge carriers confined in the nanostructure, but away from defect sites which may act as recombination centers. On the other hand, photocatalytic activity depends on separating charge carriers and delivering them to reaction centers located at the surface of the material. Consequently, a study on the photocatalytic activity of a material can provides valuable insight into energy transfer and charge carrier dynamics and their dependence on the internal structure of the CDs. In this context, we investigated photocatalytic hydrogen generation for dispersions of the CDs under illumination with a xenon lamp. A small amount of methanol (6% by volume) was added for use as a hole scavenger. While no bubble formation could be observed on short timescales, the gas phase was removed from the cuvettes after a specific time and the H_2_ production was verified and quantified with a gas chromatograph. The amount of evolved H_2_ as a function of illumination time increases linearly with time with no sign of abatement up to illumination times of over 24 h (Fig. 5a). Comparing the different CDs, the formation rate of hydrogen (i.e., slope of a curve in Fig. 5a) decreases between CD_0.04_ and CD_0.5_, but then markedly increases for samples with higher concentrations of BPEI. The highest formation rate, 18.7 µmol g^−1^ h^−1^ under full Xe lamp illumination, was measured for the CD_2_ sample. Bandpass filters were inserted into the setup to partially block the illumination light and the H_2_ production was monitored (Supplementary Fig. 6). The production rate was found to correspond strongly to the absorption spectrum of the samples, with the highest rates produced, when illuminating at the absorption maximum around 360 nm. This suggests that the same photoexcited domains responsible for the PL are also responsible for the absorption leading to H_2_ generation. The CDs appear stable under irradiation and retain their photocatalytic properties during the H_2_ production, as was further confirmed by cycling experiments in which the headspace in the reaction vessel was repeatedly evacuated at regular time intervals (Fig. 5b). The formation rate of H_2_ remained virtually the same in all cycles, for altogether an illumination time of over 30 h.

An important aspect in photocatalytic experiments is the ability to reduce water under neutral conditions. Many photocatalysts require strongly acidic or basic conditions which limits their applicability^60^. In contrast to these, the CDs actually exhibit their highest H_2_ generation nearly neutral pH (7.5–8.5), as shown for the case of the CD_2_ sample (Fig. 5c). Similar behavior was also seen for the CD_0.04_ and CD_0.5_ samples (c.f. Supplementary Fig. 7). This makes the CDs even more suitable for future applications. The exact mechanism of the proton reduction on the CDs is yet to be elucidated, however it appears that full protonation or deprotonation of the surface amine groups is detrimental to the reaction rate. Interestingly, the H_2_ generation reaction was found to proceed even if no additional hole scavenger was added, albeit with a reduced rate (Fig. 5d). It is possible that in the absence of an additional sacrificial agent, the amine groups in the *sp* ^3^-hybridized matrix act as an internal hole scavenger^61^. This is in agreement with common use of amines in this role in photocatalytic experiments^62^. An increased amount of hole scavenger only slightly increased the H_2_ generation rate, so the 6% value produces close to the maximum efficiency. Importantly for this reaction, no additional co-catalyst, metal or otherwise, was used in the experiments. This means that the measured photocatalytic activity reflects the intrinsic capacity of the reaction centers in the CDs. To quantify the photocatalytic efficiency of CD_2_, the quantum efficiency of hydrogen generation (QE) has been calculated at full xenon lamp irradiation and illuminated through a 340 ( ± 15) nm bandpass filter (Supplementary Methods and Supplementary Fig. 8). Calculated QE values are 0.84 % for CD_2_ illuminated through the 340 nm band pass filter (Supplementary Table 1), which is comparable with other carbon-based material systems that similarly do not require a co-catalyst^12, 63^.

## Discussion

**Fig. 6:**
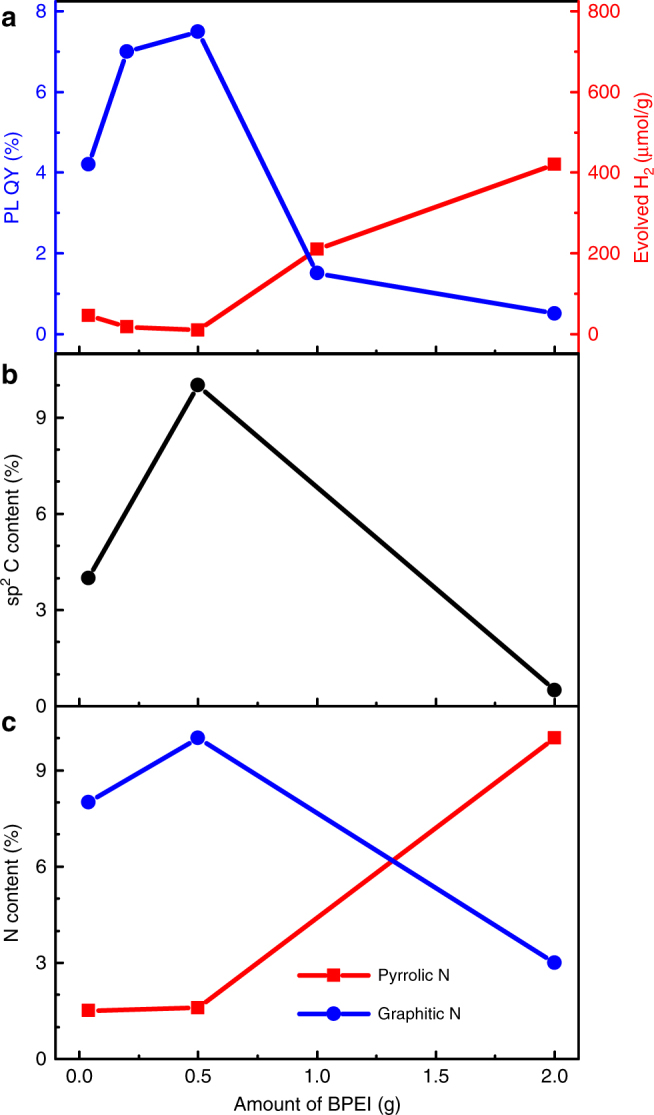
Effect of BPEI content on the properties of CDs. **a** Dependence of the hydrogen evolution and photoluminescence QY on the BPEI content in CDs. **b** *sp* ^2^ carbon content and **c** nitrogen atom incorporation modes as functions of concentration of BPEI

The inverse relationship between the PL QY and the photocatalytic H_2_ generation suggests at first a trade-off mechanism between these two pathways for the photoexcited charges (Fig. 6a). Such an anti-correlation between the two processes in not uncommon and is usually explained in terms of the photocatalytic process constituting an additional and competitive non-radiative channel^64^. However, there have also been reports of a positive correlation between the two processes. In this case, it is considered that instead of a mutual competition, they both benefit from common factors leading to a decrease in the dominant non-radiative recombination rate, for instance a reduction in the number of defect sites^65^. In this paper, the quantum efficiency of either process does not exceed 10%. Hence, not only the tradeoff between PL and photocatalysis but—more especially—the competition with the dominant non-radiative processes needs to be taken into consideration. The XPS results (Fig. 6b, c) reveal a striking agreement between the carbon content of the aromatic domains and the graphitic nitrogen content with the trend of PL QY. Graphitic nitrogen is perfectly compatible with the *sp* ^2^-hybridized polycyclic hydrocarbons embedded in the *sp* ^3^ matrix. It substitutes a carbon atom, providing an additional electron to the π^∗^ orbital in the process^32^. This delocalized electron is considered to increase the radiative lifetime of the aromatic domains^53, 66–68^. Moreover, the graphitic N enhances the rigidity of the domains, which substantially reduces the probability of non-radiative relaxation through vibrational modes of the molecules^31^. Such *sp* ^2^ flakes tend to have very few atomic defects which might act as charge carrier traps and recombination centers^69^. Altogether these effects manifest themselves in a decrease in the non-radiative recombination rate and thereby explain the enhanced PL QY observed for samples with the highest content of *sp* ^2^ carbon and graphitic nitrogen atoms (i.e., CD_0.5_).

For higher BPEI concentrations, the amount of nitrogen atoms incorporated into the *sp* ^2^ domains in the graphitic configuration does not increase further. Quite the contrary happens, as shown in Fig. 6b, with the N atoms found predominantly at the edges of the aromatic domains in the pyrrolic, as well as—to a lesser extent—in pyridinic positions and pendant amine groups. This has several important implications. First, the rigidity of the structures is slightly compromised allowing for more vibrational modes and thereby a stronger non-radiative recombination channel and facilitated energy/charge transfer towards the exterior defect sites^42, 70^. Second, the pyrrolic nitrogen with a higher electron affinity than the neighboring carbon atoms and other aromatic nitrogen atoms attracts the π-electrons toward the pentagonal edge site of a polycyclic aromatic molecule. This enables efficient interfacial electron transfer processes^71, 72^, which is crucial for the photocatalytic activity. The beneficial role of edge-coordinated N atoms for the catalytic efficiency has in fact been demonstrated in several reports^43, 47, 73^. In particular, graphitic and pyrrolic nitrogen appear to act in synergy, where the former serves as an electron transfer mediator, whereas the latter provides the catalytically active sites^71^. Finally, the unreacted amine groups remaining from the precursor are expected to function as hole traps, facilitating charge separation and hole transfer. The latter is often considered to be the limiting step in photocatalytic hydrogen generation^60, 74^. In this context, analysis of the internal structure and optical properties of the CDs provides a sound explanation of the strongly enhanced photocatalytic activity and also a reduced PL QY of the CD_2_ sample. Interestingly, there is no simple trade-off mechanism, but rather a common reason traced to the roles of graphitic and pyrrolic N atoms for enhancing PL against the non-radiative processes, while also decreasing the photocatalytic activity. The same applies to the vice versa process where an increase in the photocatalytic rate at the expense of the non-radiative processes has a parallel effect of decreased PL QY.

In summary, we report a control of a dominant functionality of CDs prepared from citric acid and BPEI through variation of the internal structure. Specifically, we show that changing the BPEI content during the synthesis determines the inclusion mode of nitrogen atoms in the aromatic domains of the CDs. At intermediate levels of BPEI, numerous *sp* ^2^ domains grow with graphitic nitrogen which allow for high PL QY. At high (and very low) BPEI content, the N atoms are located primarily at the edge sites of the aromatic domains which attract the electrons, enabling their further transfer and thereby leading to an enhanced photocatalytic activity. This system demonstrates the versatility of CDs, which can be tuned between photoluminescent and catalytic properties through very simple synthetic means.

## Methods

### Materials

Citric acid (ACS reagent ≥ 99.5%), branched polyethylenimine (BPEI M_n_~ 600 by GPC) were purchased from Sigma Aldrich and used as received without further purification. Milli-Q (type-I, ultrapure) water has been used for the synthesis and the dilution steps in preparation for the characterization. HPLC grade methanol (Sigma Aldrich, purity ≥ 99.9%) was used as a hole scavenger in H_2_ generation experiments.

### Synthesis of citric acid derived N-doped carbon dots

Aqueous soluble N-doped CDs were prepared by a simple microwave-assisted pyrolysis technique described elsewhere^48^. Briefly, 1 g of citric acid was solubilized in 20 ml water, followed by an addition of BPEI. We have varied the amount of BPEI from 0.04 to 2 g, while the citric acid amount remained constant (1 g). The reaction mixture was stirred under 1200 rpm at 65 ^°^C for 10 min. Next, this solution was transferred to a 250 ml conical flask and the volume of the mixture was adjusted with water to 50 mL. The flask was then kept in a simple domestic microwave for pyrolysis at 900 W output power for 35 min. This was performed in five steps of heating, each lasting 6 min with 1 min gap between the steps. During this process the volume was kept constant by addition of water after each pyrolysis/heating step. After the final step, the solution was cooled down to room temperature (25 °C) and centrifuged for 10 min at 12,000 rpm to remove the insoluble product. The brownish supernatant containing N-doped CDs was then purified by a filtration with 0.45 μm syringe filter. Finally, to remove the unreacted product, a dialysis has been performed by using the dialysis membrane having a molecular cut-off at 2000 Dalton.

### Morphological and optical characterizations

Transmission electron microscopy (TEM) and high resolution transmission electron microscopic (HR-TEM) images have been taken by Titan Themis at 300 kV accelerating voltage. The sample was prepared by air-drying an aliquot of the aqueous solution of CDs on a carbon coated copper grid. Raman spectroscopy has been performed on a drop casted film of CDs on a thin glass slide by using a 632.8 nm laser source at its full power (Melles Griot 05-LHP-991) coupled with a spectrometer (Princeton Instrument Acton SP2500) and a microscope (Zeiss Axio Scope. A1) for laser beam alignment. To avoid the effect of the source laser on the Raman signal we have used a 632.8 nm max line laser clean-up filter (Semrock LL01-633-12.5). The chemical composition of the samples was measured using an X-ray photoelectron spectrometer (XPS) equipped with a VSW TA10 X-ray source and a VSW HA100 hemispherical analyzer. A VSW AS10 argon ion gun was used to remove the surficial layers of the sample. Each sample was measured before and after sputtering for 15 min at 1 kV and an ion current of 7.5 μA. UV–vis absorption measurements were taken with a Varian Cary 5000 UV–vis-IR spectrometer. Photoluminescence (PL) spectra were recorded with a Horiba Jobin Yvon Fluorolog-3 FL3-22 spectrometer equipped with a 450 W Xe lamp, double monochromators for both excitation and emission and a water-cooled Horiba R928 photomultiplier tube mounted at 90° angle. The obtained spectra were corrected for the spectral sensitivity of the detector and divided by the excitation intensities. To perform time-resolved fluorescence measurements, an NKT SuperK EXTREME EXR-20 white light laser equipped with an EXTEND-UV box running at 350 nm with a repetition rate of 5.56 MHz was used to excite diluted CD solutions. The signal was detected with a Princeton Instruments monochromator fiber-connected to a low-noise Avalanche Photodiode from Excelitas. A PicoQuant TimeHarp 260 card was used for processing the signal.

### Photocatalytic hydrogen generation

The experiments were performed in a custom built metal-free cuvette with Teflon-lined septum on top under argon atmosphere. The cuvette was filled with 3 ml of aqueous solution containing 5 mg CDs and 6 % methanol (v/v). A 450 W Xe lamp without any filter provided the light illumination. Furthermore, several band pass filters (280 nm, 320 nm, 340 nm, and 356 nm with FWHM ~25 nm) and two long pass filters (390 nm and 420 nm, respectively) were used in control experiments at selected wavelengths. The illumination area was 1 cm^2^ in each case at intensity of 600 mW cm^−2^. The amount of evolved H_2_ has been determined at regular intervals by taking 10 μL aliquots out of the headspace above the dispersion of the reaction cuvette and injecting them into a Shimadzu GC 2014 gas chromatograph running with argon as a carrier gas.

### Data availability

Data is available from the corresponding author upon reasonable request.

## Electronic supplementary material

Supplementary Information
