# Supplementary material for: Effect of nitrogen atom positioning on the trade-off between emissive and photocatalytic properties of carbon dots
Source: Nat Commun. 2017 Nov 9;8:1401. doi: 10.1038/s41467-017-01463-x (PMC5680170; doi:10.1038/s41467-017-01463-x)
Supplement: Supplementary file 1 — Supplementary Information [file 41467_2017_1463_MOESM1_ESM.pdf]

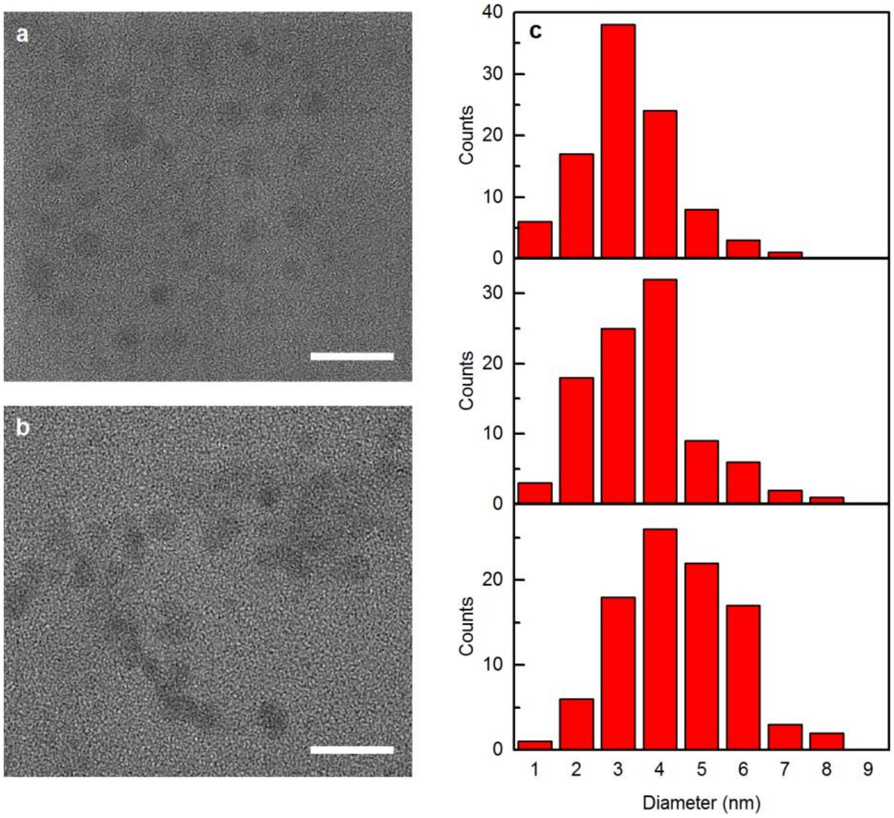

Supplementary Figure 1: TEM images of (a) $CD_{0.04}$ and (b) CD_2 (scale bar 10 nm). (c) Size distribution of different CDs (top: 0.04g, middle: 0.5g, bottom: 2g).

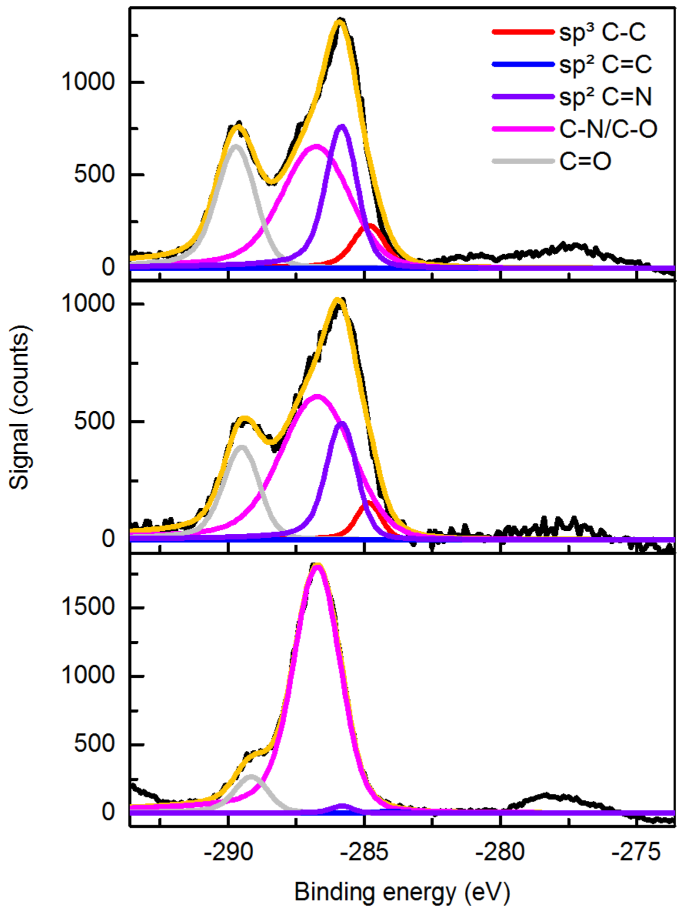

Supplementary Figure 2: C1s XPS spectra of CDs with varying BPEI content (top: 0.04g, middle: 0.5g, bottom: 2g) taken prior to argon ion sputtering.

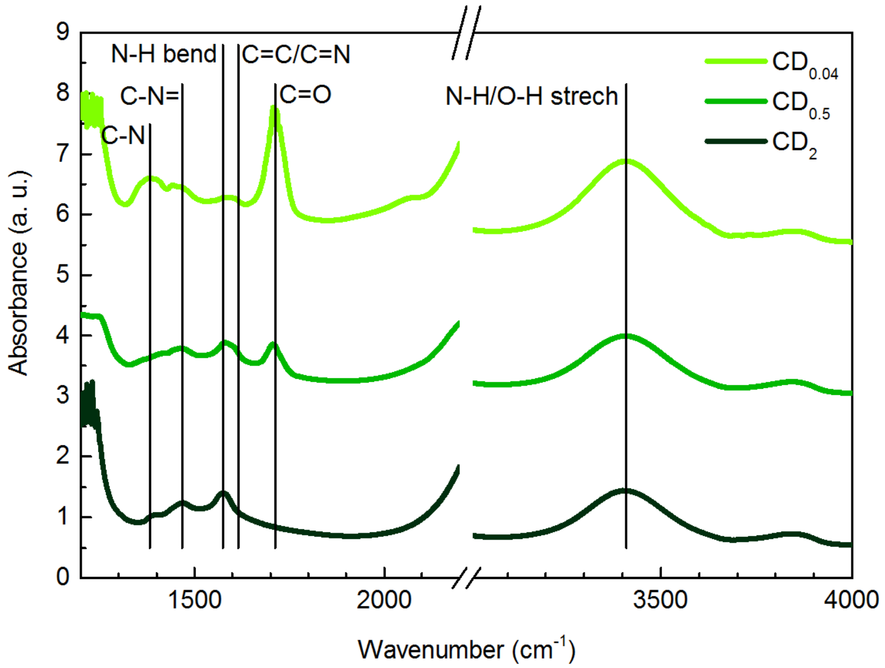

Supplementary Figure 3: FTIR absorption spectra of $CD_{0.04}$, $CD_{0.5}$ and CD_2 in D_2O (Details are in Supplementary Notes).

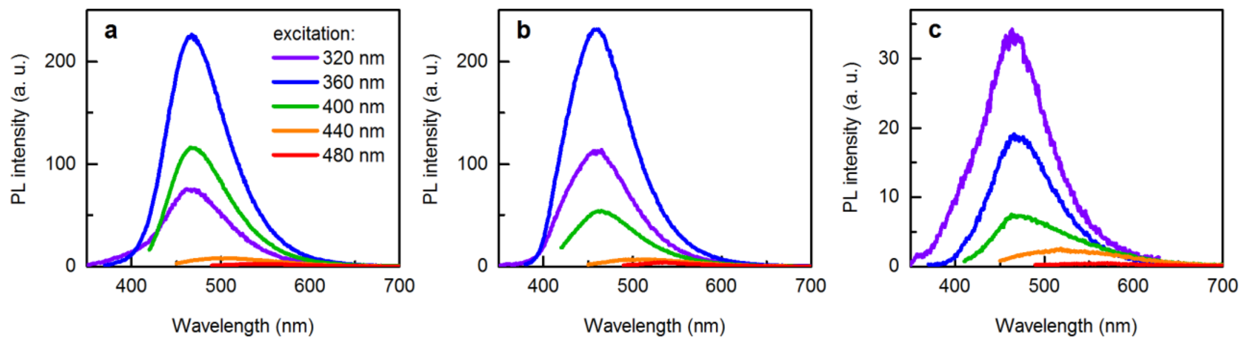

Supplementary Figure 4: Excitation-dependent PL spectra of (a) $CD_{0.04}$, (b) $CD_{0.5}$ and (c) CD_2.

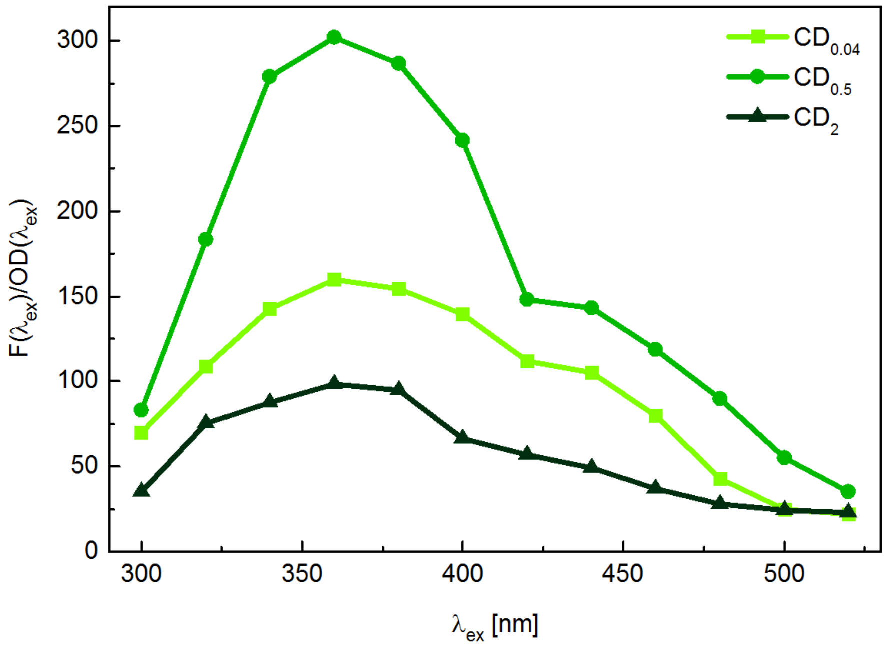

Supplementary Figure 5: Plot of integrated PL area (divided by optical density) versus excitation wavelength for different CDs.

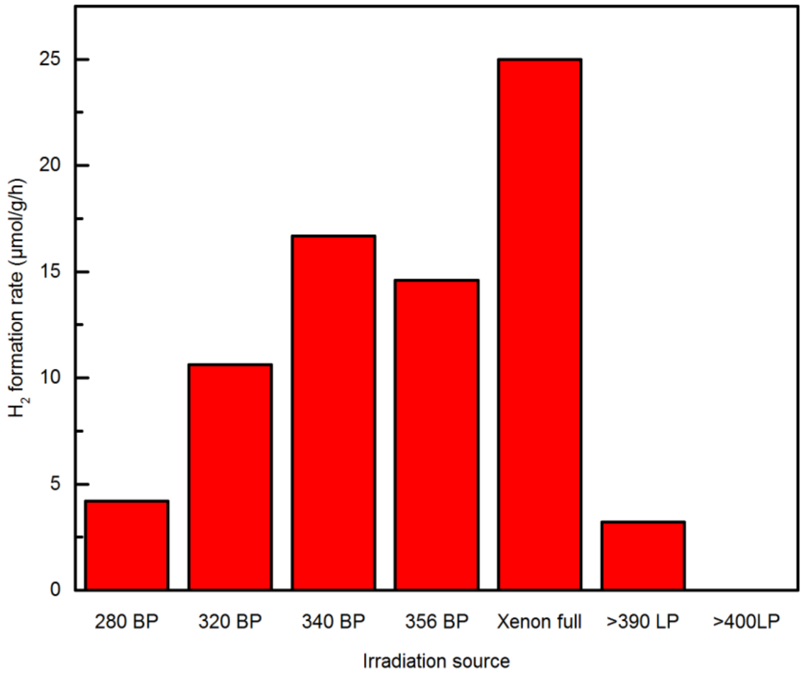

Supplementary Figure 6: Hydrogen generation rate as a function of excitation wavelength using a Xenon lamp and excitation filters. BP denotes use of a bandpass filter (average 20-25 nm width), LP denotes use of a long-pass filter.

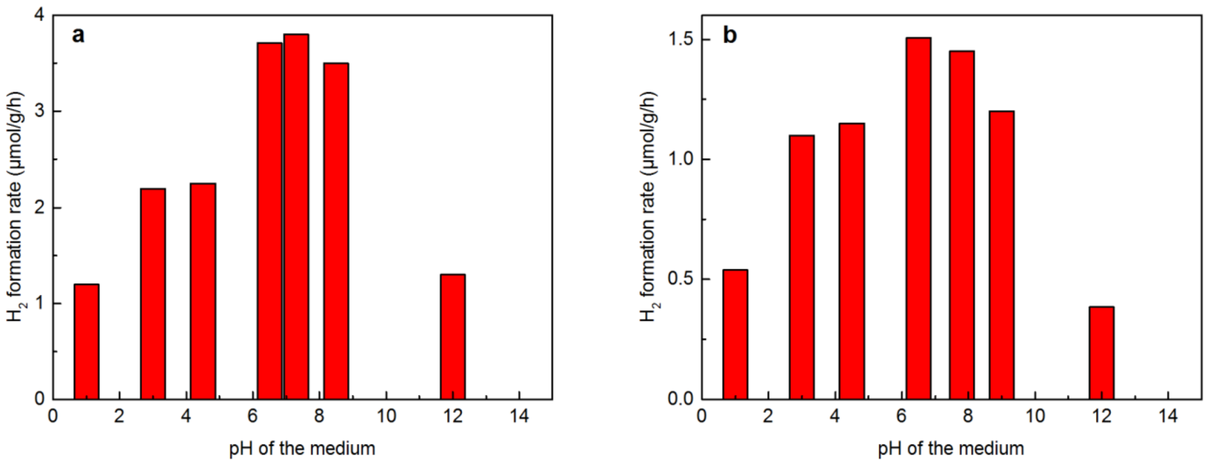

Supplementary Figure 7: Hydrogen formation rate for (a) $CD_{0.04}$ and (b) $CD_{0.5}$ as a function of pH.

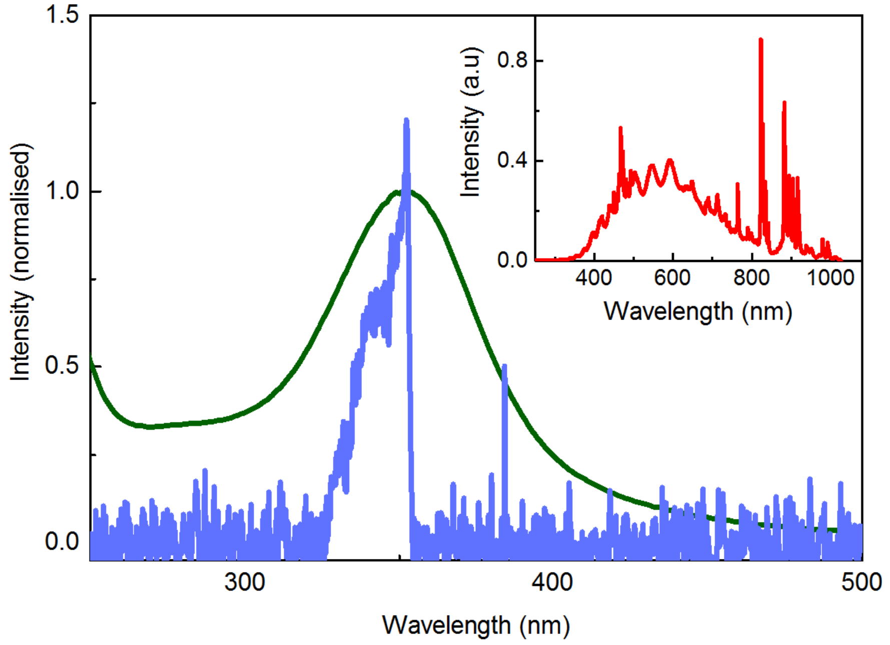

Supplementary Figure 8: Spectral overlap of the CD_2 absorption spectrum (green) and Xenon lamp spectrum filtered with a 340 nm band pass filter (blue). Inset is the full Xenon lamp spectrum.

Supplementary Table 1: Quantitative values of intensity of light, total number of photons, total number of absorbed photons, amount of hydrogen generation, photonic yield of hydrogen generation and quantum yield of hydrogen generation (detail methods are in Supplementary Methods section).

	Light intensity	Total number of photons	Total number of absorbed photons	Hydrogen generation	Photonic yield	Quantum yield
Units	mW/cm^2	$s^{-1}cm^{-2}$	$s^{-1}cm^{-2}$	µmol/h	%	%
Full spectrum	600	$1.89 \cdot 10^{18}$	$1.9 \cdot 10^{18}$	0.13	0.0023	0.10
Bandpass 340nm	4.8	$1.07 \cdot 10^{16}$	$3.18 \cdot 10^{15}$	0.08	0.25	0.84

Supplementary Note 1:

The FTIR measurements were conducted in D_2O to avoid unwanted O-H stretching from H_2O. Results show broad bands (~3200-3500 cm^{-1}) due to vibrational stretching of O-H/N-H groups for all CDs suggesting multiple N and /or O containing surface functional groups. In addition, relatively strong C=O stretching bands (~1720 cm^{-1}) have been observed for both $CD_{0.04}$ and $CD_{0.5}$, while they completely vanish for CD_2. Predominant surface functionality by –COOH groups render the surface charge negative for $CD_{0.04}$ and $CD_{0.5}$, whereas the surface charge of CD_2 remains positive due to –NH groups. These observations corroborate the Zeta potential data mentioned in the main text. Moreover, weak bands due to stretching vibrations of C=C and/or C=N (~1600-1610 cm^{-1}) bonds confirm the presence of aromatic domains in $CD_{0.04}$ and $CD_{0.5}$. These weak bands are completely masked by bending vibration of –NH groups (~1570 cm^{-1}) for CD_2. The XPS data included in the main text paints a similar picture, of a larger extent of aromatic domains for $CD_{0.04}$, $CD_{0.5}$ compared to CD_2. The presence of nitrogen-containing aromatic domains demonstrated by the XPS data was also confirmed by the FTIR measurements, which show a clear sign of stretching vibrations due to C-N= bonds (~1420-~1470 cm^{-1}). The increased presence of the N-H groups at the surface of the CD_2 sample could further explain the higher efficiency for H_2-production in this material, as amine groups at the surface could act as hole-acceptors, enhancing the photocatalytic processes and making the use of a hole scavenger unnecessary.

Supplementary Methods:

Calculation of H_2 generation efficiency:

To estimate the hydrogen generation efficiency the spectrum of the Xenon lamp itself and equipped with a 340 nm bandpass filter (Semrock FF01-340/22-25) was recorded with a Thorlabs CCS200 USB spectrometer. The intensity of the lamp with and without the filter was measured with a Thorlabs PM200 on basis of the corresponding spectra. The values were 600 mW/cm^2 without and 4.8 mW/cm^2 with the filter. Using the spectrum and the total power the power $P(\lambda)$ at each wavelength λ (in 1 nm steps) could be calculated:

$$P(\lambda) = \frac{I(\lambda)}{I_{ges}} P_{total} \qquad \text{Supplementary Equation (1)}$$

with $I(\lambda)$ being the intensity of the spectrum measured at the wavelength λ, I_{ges} the integrated intensity of the whole spectrum and P_{total} the measured intensity of the lamp. The number of photons $N(\lambda)$ emitted at a certain wavelength per cm^2 and second can then be calculated as

$$N(\lambda) = \frac{P(\lambda)}{hc} \cdot \lambda = \frac{P(\lambda)}{6.63 \cdot 10^{-34} J \cdot s \cdot 3.00 \cdot 10^{8} \frac{m}{s}} \cdot \lambda \qquad \text{Supplementary Equation (2)}$$

Since the illuminated area is 1cm^2 the total number of photons reaching the sample per second can be calculated by summing over the whole wavelength range:

$$N_{tot} = \sum_{\lambda} N(\lambda) \qquad \text{Supplementary Equation (3)}$$

The photonic efficiency for hydrogen generation can then be calculated using the hydrogen generation rate n_{H2} and the number of photons per second

$$PE_{H_2} = \frac{2n_{H_2}}{N_{tot}} \qquad \text{Supplementary Equation (4)}$$

If only the absorbed photons should be taken into account the amount of photons per wavelength *N(λ)* have to be multiplied with a factor considering the optical density A(λ). For 3 cm path length this ends up in

$$N_{abs}(\lambda) = \left(1 - 10^{-3 \cdot A(\lambda)}\right) \cdot N(\lambda) \qquad \text{Supplementary Equation (5)}$$

The quantum efficiency is then calculated as

$$QE_{H_2} = \frac{2n_{H_2}}{N_{tot_{abs}}} = \frac{2n_{H_2}}{\sum_{\lambda} N_{abs}(\lambda)} \qquad \text{Supplementary Equation (6)}$$

The values obtained from these calculations for the sample CD_2 are shown in the Supplementary Table 1.
